# Comparisons of Costs between Black Caribbean and White British Patients with Advanced Multiple Sclerosis in the UK

**DOI:** 10.1155/2014/613701

**Published:** 2014-02-05

**Authors:** Wayne Smith, Paul McCrone, Cassie Goddard, Wei Gao, Rachel Burman, Diana Jackson, Irene Higginson, Eli Silber, Jonathan Koffman

**Affiliations:** ^1^King's College London, Institute of Psychiatry, Centre for the Economics of Mental and Physical Health, London SE5 8AF, UK; ^2^King's College London, Cicely Saunders Institute, Department of Palliative Care, Policy & Rehabilitation, London SE5 9PJ, UK; ^3^King's College Hospital NHS Foundation Trust, Neurology Department, London SE5 9RS, UK

## Abstract

*Background*. Multiple sclerosis (MS) is now more common among black and minority ethnic groups in the UK but little is known about the costs of care amongst different ethnic groups. *Objective*. This study examined and compared service use and costs for people severely affected with MS from Black Caribbean (BC) and White British (WB) backgrounds in the UK and identified predictors of cost for both groups. *Method*. Population-based cross-sectional study of 43 BC and 43 WB patients with MS (EDSS ≥ 6) and their informal caregivers recruited from an MS service in southeast London. Interviews collected data on health and social service use and informal care support. Costs were calculated using UK unit cost data. Using regression analyses we compared costs between the ethnic groups and identified possible predictors of cost. *Results*. The mean (SD) costs for the WB and BC groups were **£**25,778 (**£**39,387) and **£**23,186 (**£**30,433), respectively. Results identified no significant difference in total cost between the two ethnic groups. The EDSS score alone was a significant predictor of cost. *Conclusion*. Similar costs between ethnic groups indicate that with regard to this MS service and geographical area, access to care was not affected by ethnicity.

## 1. Introduction

Multiple sclerosis (MS) is the most common neurological disease causing disability in young adults, affecting disproportionally more women than men. It is a highly varied and unpredictable condition with a poorly understood aetiology, and there is currently no cure [1]. Those affected by MS experience a range of distressing symptoms, which in the more advanced stages can include sensory and motor disturbances, fatigue, pain, bladder and bowel dysfunction, and spasticity, cognitive, and mood changes [2, 3]. Clinical symptoms, young onset, and long disease duration can all have a profound effect on quality of life. It might therefore be expected that use of health and social care services over a prolonged period of time will be elevated and therefore the costs associated with symptom management and support will be significant [4]. In addition, the costs to those with the condition and their families due to productivity loss and informal care have been identified as being substantial [5].

MS is more prevalent among people living in the United Kingdom, Europe, and North America compared to Asia and Africa [6–8]. Ethnicity is also thought to influence clinical manifestations of MS; some groups are reported to experience more aggressive disease and greater levels of disability [9, 10]. Increasing evidence indicates that a growing number of people from migrant populations to the UK are developing MS [11, 12].

To date, the financial burden of MS has been examined across a growing number of countries including the UK [13]. Trisolini and colleagues identified that the total average direct medical and nonmedical cost per person with MS in the UK in 2007 was 30,827 US dollars. It concluded that MS causes substantial economic burden on patients, their families, and society in terms of the direct and indirect costs as well as intangible costs [14]. To date, however, no research has examined the interface between health service use and costs of care for people severely affected by MS from different ethnic groups. Addressing these concerns is important for the following reasons. First, new evidence identifies more rapid disease progression and higher levels of loss and cognitive impairment among Black Caribbean (BC) people severely affected by MS compared to those among their White British (WB) peers. This is suggestive of a need for earlier identification and management of BC patients by healthcare services [15].

Second, there is increasing evidence that people from black, Asian, and minority ethnic (BAME) groups experience multiple social disadvantage and poor access to health services [16–19].

Third, increasing globalisation has brought with it large numbers of BAME people who have migrated to developed countries [20]. In 2005 alone, there were an estimated 191 million immigrants across the globe: approximately 64 million of these immigrants arrived in Europe and 44 million in North America, a tripling of the immigrant populations in these regions compared to twenty years earlier [21].

Understanding health service utilisation and costs of care across different communities is therefore critical to address concerns about social justice and to maximise opportunities for health gain. In this paper we therefore aim to (i) describe and compare the service use patterns for BC and WB patients severely affected with MS (PwMS), (ii) calculate and compare the care costs between both groups, and (iii) identify other demographic and clinical predictors of service.

## 2. Methods

Because this was part of a wider study which investigated the progression, symptoms, and psychological concerns of BC and WB people with severe MS, the setting, recruitment strategy, and data collection methods have been previously published [15]. It was thought important to outline these procedures in the current study for clarity. However, as previously mentioned, this research reports mainly on the cost of service use between the two groups and investigates the potential predictors of cost between the two ethnic groups.

### 2.1. Ethics Statement

Ethical and research governance approval were obtained from King's College Hospital Research Ethics Committee (South London REC Office (2) (ref:10/H0808/43)) and three Research & Development Departments (Lambeth, Southwark, Lewisham & Bexley RDLSLBe545, Guy's & St. Thomas RJ112/N042, and South London Healthcare SLHT/2010/UCSN/neuro/27).

### 2.2. Design

This was a population-based study, using a face-to-face quantitative survey.

### 2.3. Setting

This study took place in the existing MS services in southeast London, a partly materially and socially deprived geographical area with a total population of 1.61 million people and with one of the highest concentrations (25%) of BC people in the country [22]. A comprehensive MS consultant-led service covers the southeast London boroughs with a network of MS nurses based in both hospitals and the community. The majority of people with MS in this geographical area are cared for by the MS specialists at King's College Hospital NHS Foundation Trust either at King's College Hospital itself (around 500 people with MS) or within services located at other district general hospitals (around 1000 people with MS). In addition there are six community MS nurses working in each of the southeast London boroughs.

### 2.4. Participants

#### 2.4.1. Inclusion Criteria

Adults aged 18+ years, a confirmed diagnosis of MS based on the modified “McDonald Criteria”—guidelines of recommended diagnostic criteria for multiple sclerosis [23] and, an Expanded Disability Status Scale (EDSS) [24] score of ≥6.0 (meaning that they require unilateral support to walk a distance of 100 m or more and where higher scores are indicative of greater levels of MS disability). Subjects were self-identified as WB or BC and required sufficient cognitive capacity to provide informed consent and complete the interviews. Capacity was determined by referring clinicians and evaluated using the blessed orientation memory concentration test (BOMCT), where scores of 15 and over indicated increased cognitive impairment and thereby diminished capacity [25].

#### 2.4.2. Exclusion Criteria

Patients not providing consent or lacking capacity.

### 2.5. Recruitment and Informed Consent Procedures

Potential study subjects from both ethnic groups were informed of the study by their neurologist or MS specialist nurse. In addition in order to maximize BC recruitment, information about the study was passed through a voluntary sector organisation in the region for black patients.

At this point eligible PwMS were provided with a comprehensive study participant information sheet and given 48 hours to consider their involvement in the study. Those who expressed an interest in the study were then contacted to organise a convenient time and location (usually the patient's home) for a face-to-face interview. Written informed consent was provided prior to beginning each interview. In order to eliminate any potential for coercion potential participants who declined to participate in the study were reassured verbally and in writing that their current/future treatment and care would not be compromised in any way.

#### 2.5.1. Data Collection: Service Use and Cost Measurement 

Data were collected from participants during a face-to-face interview. These interviews took place between June 2010 and October 2011. The Client Service Receipt Inventory [26] (CSRI) was used to collect data on the use of health (primary and secondary care) and social care by participants in the six months prior to interview. The CSRI attempts to capture all information on the use of services and support over a particular period, regardless of whether this was MS related or not.

Hours of care received from families/friends for help in specific areas (personal care, help with medical procedures, help both inside and outside the home, and time spent on call) were also reported. Service costs for each category were estimated by combining these data with appropriate unit cost information. For example, neurology outpatient cost was calculated as follows: number of outpatient visits multiplied by the mean (unit) cost per neurology outpatient visit. Cost of a clinician visit was calculated as time spent with a health professional multiplied by national cost per hour of care multiplied by number of visits. Unit costs for hospital care and investigations were taken from the UK NHS Reference Costs database 2010-2011 [27]. Other costs were obtained from the annual unit costs compendium produced by the University of Kent [28]. This provides costs for a range of activities services provided in the community and costs for some hospital activities.

Informal care is unpaid and here the value was assumed to be equivalent to the cost of a homecare worker. Sensitivity analyses were conducted to determine the effect of varying this cost on the overall costs. Where it was known that a service had been used but the quantity/contact duration was missing, the median values from other patients who had this information were used. The maximum amount of informal support that patients could indicate that they received was capped at 168 hours per week calculated as 24 hours care for seven days per week.

Information was documented on all medication taken during the six-month period. Recognised disease modifying drugs for MS (interferon beta 1a and copaxone) were costed as well as frequently used drugs for neuropathic pain (carbamazepine, gabapentin, and amitriptyline). Drug costs were calculated using prices contained in the British National Formulary (BNF) [29].

### 2.6. Analysis

The analysis reports across and compares participants' demographic and clinical characteristics between both ethnic groups.

Student's *t*-test for interval data and chi-squared test or Fisher's exact where appropriate for categorical data was used to check for significant differences in demographic and clinical characteristics between the BC and WB group.

Previous studies show a high correlation between EDSS scores and mean cost for people with MS [30, 31]. Therefore this correlation was tested in the current sample. A Shapiro-Wilk test for EDSS scores and overall cost showed both to have nonnormal distributions at *P* = 0.00026 and *P* < 0.0001, respectively. For this reason the association between the EDSS scores and costs was investigated using Spearman's rank test, a frequently recommended, nonparametric alternative to Pearson's correlation test [32].

EDSS scores were later grouped as 6-6.5, 7-7.5, 8-8.5, and 9 to easily observe trends between grouped EDSS scores and cost for both ethnic groups. Further Shapiro-Wilk tests for normality showed that the overall cost data for both ethnic groups had nonnormal distributions and as a result a bootstrap regression was used to compare the cost between ethnic groups. Advantages of a bootstrap regression are that it does not require distributional assumptions such as normally distributed errors and that it can provide more accurate inferences when sample sizes are small [33, 34].

A further bootstrap regression was performed comparing the overall cost between ethnic groups while controlling EDSS score, age, type of MS, gender, index of multiple deprivation (IMD) [35], and number of years with MS. The IMD covers domains such as housing, geographical access to services, employment, income, health, and disability, all of which are likely to be associated with disparities in health care [36]. The literature also states that genders as well as age are contributing factors to disparities in health care [37, 38]. EDSS score, type of MS, age, and length of time with disease were also included as possible clinical confounders.

### 2.7. Sample Size

It was estimated that we would need to recruit at least 39 participants in each group to detect clinical differences in MS symptoms [15]. Because this research focuses on patients with advanced MS (EDSS > 6) it was difficult to recruit suitable patients especially from within the BC group which is an emerging but growing population in the UK. A study by Kobelt and colleagues found the distribution of people with EDSS score ≥7 to be 19% [31].

## 3. Results

We approached 97 PwMS of whom 46 were BC and 51 were WB PwMS. 43 BC and 44 WB patients agreed to participate. One person in the WB group was excluded after scoring over 15 with the BOMCT. Reasons for declining participation included those whose illness progressed and became too unwell to interview, complex family circumstances, and those who did not express an interest in the survey.

Most participants were born in the UK, apart from nine in the BC group (Table 1). Over two-thirds were women. BC participants were typically younger (mean ages, BC 47.7 yrs, WB 57.5 yrs, *t*-test = 3.97, *P* < 0.001). There was a relatively even distribution of MS disease types across the whole sample. Of the 86 participants recruited, MS disease types were relapsing remitting (*n* = 24), secondary progressive (*n* = 32) and primary progressive, or progressive relapsing (*n* = 30). Twice as many BC (*n* = 16) than WB (*n* = 8) were diagnosed with relapsing-remitting MS. WB participants were diagnosed with MS for longer periods than their BC counterparts (mean 16.1 yrs for WB and 10.2 yrs for BC; *t*-test = 3.01, *P* = 0.003). Fewer WB participants were taking disease modifying medication (DMT) compared to BC participants. Medication included glatiramer acetate (*n* = 3), natalizumab (*n* = 3), interferon beta therapy (*n* = 9), and unspecified (*n* = 1). Two PwMS, one per ethnic group, were recorded as receiving chemotherapy in the form of mitoxantrone, a recognized therapy for MS.

Table 2 shows the use of specific services for each group during the six-month period. Because of the small numbers of service utilisation in some areas services were grouped. For example, emergency room visits are categorised as outpatient services. Only 4 WB and 3 BC patients used emergency services, with mean number of contacts 3 and 1 contacts, respectively.Around three-quarters of each group had GP contacts (*n* = 31, 72% BC versus *n* = 43, 79% WB) and nursing staff input (*n* = 31, 72% in both groups). Around two-thirds in both groups had neurology outpatient appointments (*n* = 28, 65% BC versus *n* = 29, 67% WB). Over one-quarter of each group had contact with allied health professionals (*n* = 30, 70% BC versus *n* = 27, 63% WB). The vast majority of participants received informal care help from family/friends (*n* = 40, 93% BC versus *n* = 39, 91% WB). In terms of numbers using services there were no major differences between the two groups. Of those who did receive care from nurses, allied health professionals, and informal care, the number of contacts was greater for the White British group.

The most expensive formal services were home help and inpatient services, and these were both greater for the BC group (mean cost and standard deviation: *£*3,112 (*£*6,378) BC versus *£*1,960 (*£*3,546) for home help and *£*505 (*£*1,843) BC versus *£*180 (*£*1,004) WB). The higher cost of home help care for the BC group is in spite of similar numbers of contacts and this suggests that the duration of contacts for home help was greater in this group.

When the UK minimum wage of *£*6.19 was used to value informal care the mean overall cost per PwMS was *£*10,290 for the BC group and *£*9,789 for the WB group. Replacing the minimum wage rate with the median UK hourly wage *£*12.76 increased the mean costs per PwMS to *£*16,175 and *£*16,615, respectively. For final evaluations informal care cost was valued the same as a homecare worker. The total mean (SD) six-month cost was *£*17,188 (*£*28,567) for BC PwMS and *£*22,415 (*£*38,979) for WB PwMS. When stratified by gender there was a higher mean cost in both ethnic groups for males compared to females. The mean cost of medication per patient for BC patients was *£*1,338, almost five times as much as for WB patients (*£*273).

**Figure 1:**
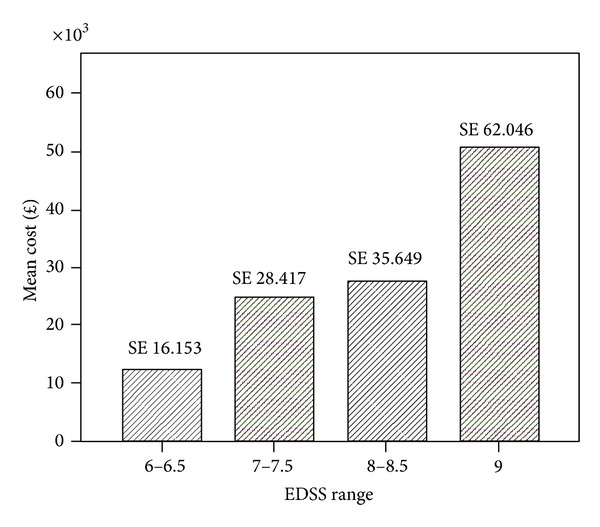
Mean cost and standard errors (SE) in each EDSS group for sample *n* = 86.

**Figure 2:**
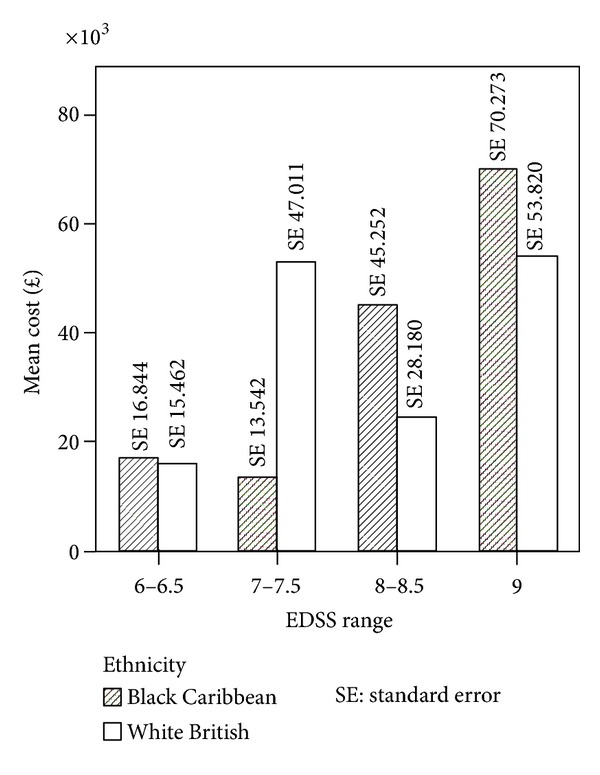
The mean cost in each EDSS group for Black Caribbean and White British participants.

**Figure 3:**
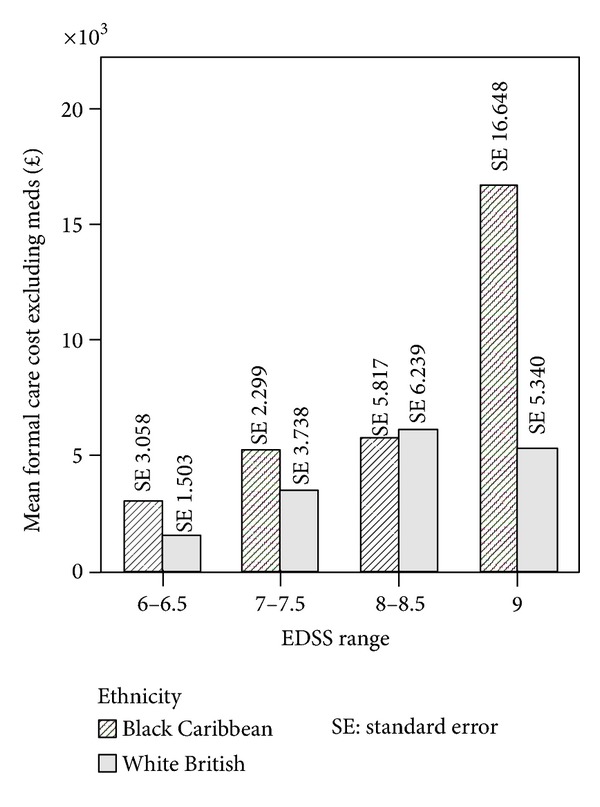
The mean formal care cost in each EDSS group for Black Caribbean and White British participants.

**Figure 4:**
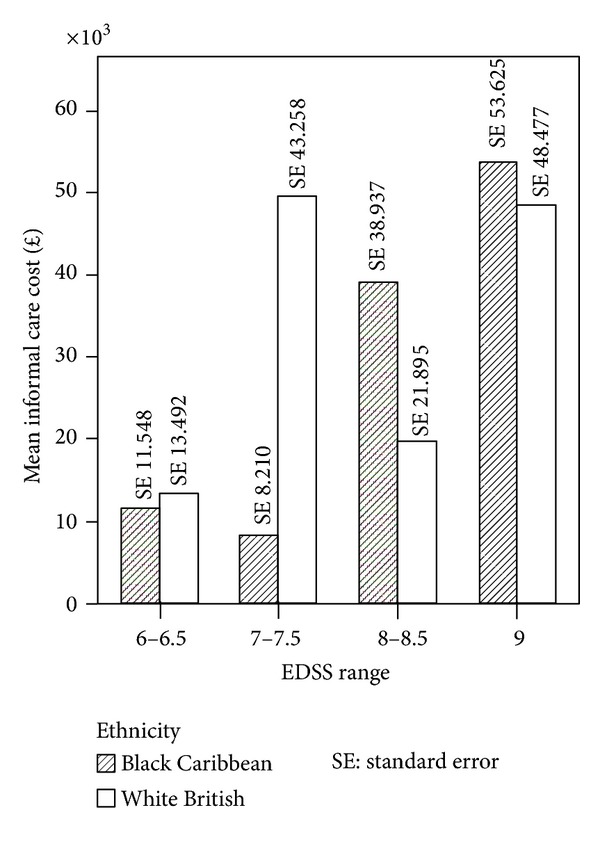
The mean informal care cost in each EDSS group for Black Caribbean and White British participants.

**Figure 5:**
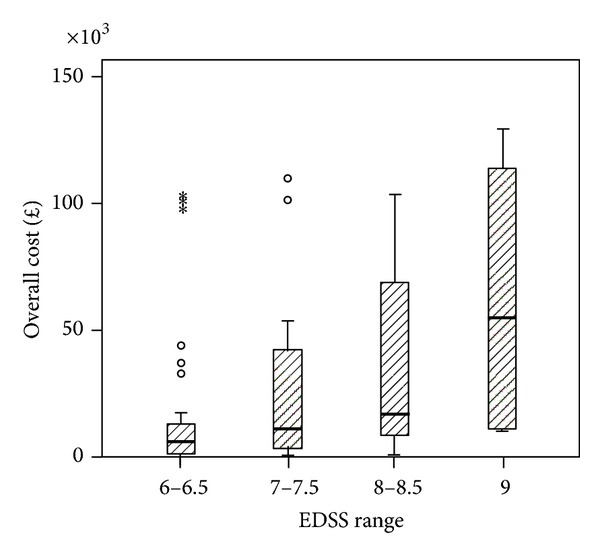
Box plot of overall costs for sample *n* = 86.

**Figure 6:**
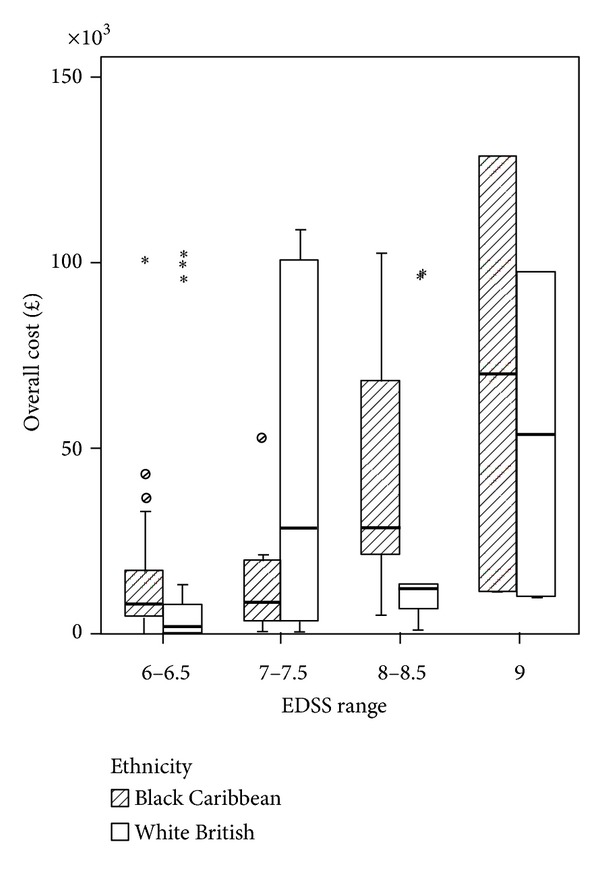
Box plot of overall costs for Black Caribbean and White British groups.

**Figure 7:**
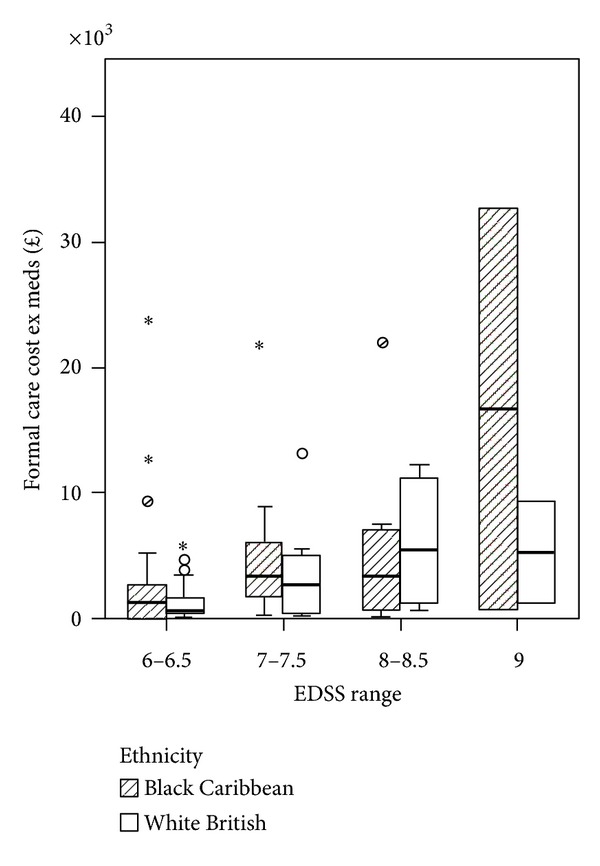
Box plot of formal care costs for Black Caribbean and White British groups.

**Figure 8:**
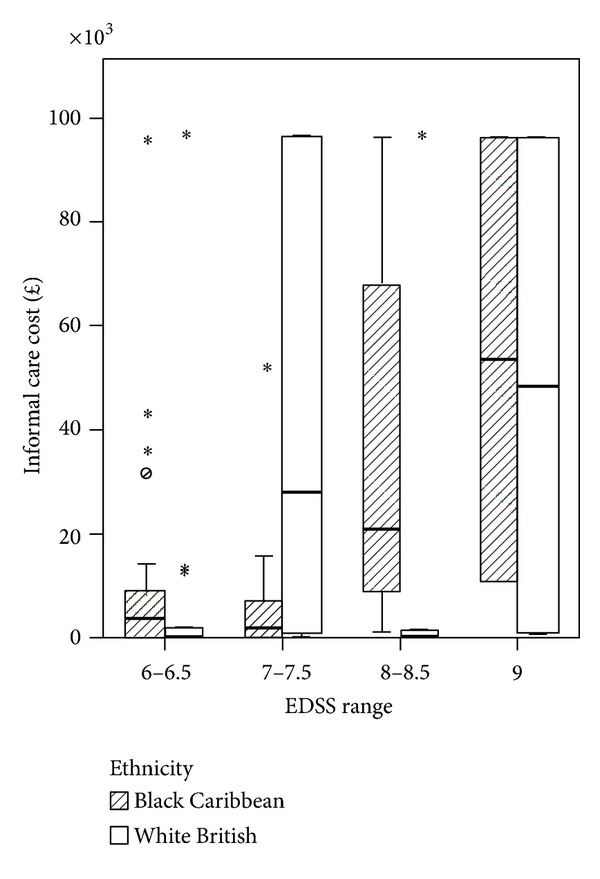
Box plot of informal care costs for Black Caribbean and White British groups.

The Spearman's rank correlation coefficient between EDSS score and cost was 0.348, significant at *P* = 0.001 for sample *n* = 86. Within the BC and WB groups the correlation coefficients were 0.307 and 0.375 and statistically significant for both groups at *P* < 0.05. By grouping EDSS scores (6-6.5, 7-7.5, 8-8.5, and 9) for all PwMS *n* = 86, we observed that costs similarly increased with disease severity (Figure 1).  Figure 2 shows the results for each ethnic group. In the BC group the higher cost is observed in EDSS group 8-8.5 and group 9 whereas in the WB sample higher costs are observed in group 7-7.7 and group 9.  Figure 3 shows the mean cost of formal care for WB and BC PwMS in each EDSS group.  Figure 4 shows the informal care costs for WB and BC PwMS in each EDSS group. Figures 5, 6, 7, and 8 are box plots depicting the spread of the cost data in relation to Figures 1, 2, 3, and 4.

A bootstrap regression with the mean cost as the dependent variable and ethnicity as the independent variable revealed no statistical difference in costs between the two ethnic groups. BC participants had costs that were on average *£*2,592 less than those for WB participants (95% CI, −17076 to 11755) (Table 3). The results of including additional independent variables (EDSS score, type of MS, age, gender, IMD score, and number of years with MS) in the bootstrap regression are included in Table 4. It shows that EDSS score alone significantly predicted cost. As the EDSS score increases by one unit the mean cost increases by *£*5,985 (95% CI, 397 to 11,992).

Although not significant, there was a negative correlation between age and overall cost of MS. Tyas and colleagues [39] also found a similar association between age and cost.

There was also a negative but nonsignificant correlation between cost and time with the disease. Tyas also proposes that at high EDSS states greater cost may be due to indirect costs and found indirect costs to be the greatest cost category. In our current study no indirect costs were included (only direct medical and direct nonmedical costs). This could account for the decreasing cost with increasing time with MS. As formal health care costs are likely to be greater in earlier years (partly due to disease modifying drugs which are recommended to be stopped in the more advanced stages of MS) after which there is an increase in social care and indirect costs. Lack of data collected on indirect cost does not allow direct cost to be offset by indirect costs over increasing time with MS.

## 4. Discussion

This population-based cross-sectional study has four main findings.**   **First, the mean cost per PwMS did not differ significantly according to ethnic group. This could be attributed to the efficient running of the service and a focus on individual patient care support depending on needs and guidelines which help to support equity in service delivery [40].

Second, there was a significant difference in the mean cost of medication between the two groups. Medication costs for the BC group were almost five times those for the WB group. The difference in drug cost is likely to be due to the disease modifying medication (DMT), where fewer WB patients were found to be taking DMTs. The BC group had more aggressive disease progression and more relapsing MS [15].

Third, informal care accounted for approximately 87% of the total cost for the WB group and 74% of the total cost for the BC. Interestingly though, the greater informal care cost for WB participants was offset by the higher cost of home help care in the BC group. Overall, the high informal care costs suggest a substantial dependency on family members in MS patients. The relatively low hospital costs may be due to a good system with close links to community services, thus preventing unnecessary hospital and inpatient care due to complications.

Fourth, EDSS scores and time with disease from diagnosis to follow up were significant predictors of costs. The finding that costs were positively associated with symptom severity supports earlier work done by Kobelt et al. [41] who also suggest that the total mean cost for patients with MS is related to the level of disability, while the latter is inversely related to utility scores measured using the EuroQol questionnaire (EQ-5D). A systematic review of the economic burden of MS by Naci et al. [13] also finds a significant increase in cost associated with an increase in disease severity as measured by the EDSS score.

This study identifies that equitable distribution of services between ethnic groups is achievable. We believe that the structure of this service can be used as a template of “gold standard” care for people with MS for black, Asian, and minority ethnic groups in other regions of the UK.

### 4.1. Study Limitations

There are several limitations to our study. First, differences in racial identity between the research participants and interviewer may potentially affect the accounts of participants' illness [42]. Matching interviewer and participant on characteristics such as ethnicity, gender, age, and social class may build rapport [43]. Despite this we were successful in recruiting and interviewing PwMS, in the more advanced stages of diseases, across both ethnic groups. While most MS studies on ethnicity rely on data from clinical records [9, 44], we collected assessments directly from patients. Structured questionnaires and specifically the CSRI have been used in the past to collect data directly from patients on the use of services [5, 45, 46]. Previous studies examine the use of patient reported questionnaires versus clinical records [47, 48]. One advantage is that you are able to obtain a broad perspective on cost using patient reported data. For example patients can report on areas such as informal care and productivity loss due to time off work.

Second, those who declined to participate or were excluded based on the aforementioned criteria may have been different from the study sample thereby introducing selection bias. Kho and colleagues support that written informed consent can potentially threaten the validity of study results because of differences between participants and nonparticipants [49]. Perhaps one way to more accurately capture costs for persons with advanced MS would be through proxy interviews from family members or carers. However the relationship of the proxy to the patient may influence the accuracy of the proxy responses and may also be affected by the opportunity for direct observation [50].

Third, the health-related measures may not have cross cultural equivalence [51]. This would assume a common understanding between BC and WB participants in relation to the questions asked. The charges of ethnocentricity can be reduced by examining an instrument's performance and acceptability within a new population. Although many of the tools we used have been heavily used among other cultural groups, none have specifically been explored within BC PwMS.

Fourth, data collection may have been subject to recall bias. PwMS were asked to recall details of resource use over a six-month period. In our study we believe that this limitation applies to both ethnic groups. Whilst previous studies have revealed such methods to be reasonable [52, 53] we are, nevertheless, mindful that alternative strategies where there has been a suggestion of ethnic differences in recalling salient facts have been included amongst other issues asking white patients to specify the month of initial symptoms and asking African American patients about the season when onset occurred [44].

Fifth, costs incurred from loss of production due to time off work, reduced productivity in work or early retirement were not included in the analyses. These costs may potentially differ between the ethnic groups.

Sixth, insufficient data was collected for us to examine whether there were differences in costs between settings and therefore it could not be included in the multivariate analysis. There may also have been differences in the quality of care received between the settings.

Finally, it is challenging to recruit individuals from some ethnic minority groups. Inclusion of minority groups in research is important and can identify clinical differences in the manifestation of diseases and also helps to address equity issues in the delivery of health care. Barriers to recruitment include lack of time, travel commitments, the stigma associated with the disease area, and a perceived financial cost to the participant. A key factor that helps to improve participation is forming a trusting relationship [54].

## 5. Conclusions

This is the first population-based study comparing BC and WB people severely affected by MS in terms of disease progression in relation to the costs associated with MS.

Our findings identify that people severely affected by MS have highly complex needs which are associated with increasing service use and informal support and have considerable costs to the health service and society. This current study supports previous studies with similar findings of high cost and that highlight the economic burden associated with MS.

Our findings show that costs between the BC and WB group were not significantly different. Therefore there was no evidence of inequitable resource allocation. As the BAME population with MS is growing it is important to review how these services are run and to ensure that all groups receive access and support appropriate to their needs. Healthcare services need to be transparent in demonstrating equitable distribution of healthcare and providing evidence in support.

Similar research methods can be used to compare MS disease progression and cost amongst other BAME groups or between BAME and white populations. This research method may be useful to Public Health England (PHE), an executive agency of the department of health where one of the primary aims of the agency is to reduce health inequalities [55]. This method may also be applicable to other areas where there may be claims of inequitable distribution of healthcare and can be extrapolated beyond the UK healthcare system to prove or disprove claims. In the United States there are often claims of these ethnic disparities. For example, Fiscella and colleagues [56] cite several examples of disparities in health service use between blacks and whites and also between other minority ethnic groups such as Latinos and Asian Americans compared with whites.

The UK National Health Service is a publicly funded healthcare system which is free at the point of service. There are a number of national policies and regulations in place that outline equity of service, measure the NHS performance, and help to improve or maintain standards of care [57–59]. These may have contributed to our findings for no evidence of inequitable allocation. Further research can examine the quality of care between settings in the NHS for people with MS or examine and compare a similar study within a different health care system, for example, the US.

## Figures and Tables

**Table 1:** Demographic and clinical characteristics of Black Caribbean and White British participants included in the study [15].

	White British (*n* = 43)	Black Caribbean (*n* = 43)	*P* value for statistical test
Place of birth:			
United Kingdom	43 (100%)	34 (79%)	Fisher's exact
Caribbean	0	9 (21%)	*P* = 0.003
Gender:			
Male	15 (35%)	11 (26%)	Chi-sq 0.88 (df = 1),
Female	28 (65%)	32 (74%)	*P* = 0.35
Mean age, SD (range) median	57.5 yrs, 12.1 (35–88) 57.7	47.7 yrs, 10.8 (27–75) 48.1	*t*-test = 3.97, *P* < 0.001
Highest level of education attained:			
Did not go to school	1 (2%)	0	Fisher's exact *P* = 0.053
Primary school	1 (2%)	4 (9%)	
Secondary school (GCSE)	13 (30%)	20 (47%)	
Secondary school (A-level)	8 (19%)	10 (23%)	
University	20 (47%)	9 (21%)	
Degree/equivalent professional qualification:			
Yes	23 (53%)	9 (21%)	Chi-sq 9.75 (df = 1), *P* = 0.002
No	20 (47%)	34 (79%)	
Employment status			
Employed full time	1 (2%)	4 (9%)	Chi-sq 0.314 (df = 8), *P* = 0.005
Employed part time	4 (9%)	0 (0%)	
Self-employed	1 (2%)	0 (0%)	
Unemployed	6 (14%)	11 (26%)	
Retired (because of age)	15 (35%)	2 (5%)	
Retired (because of illness)	15 (35%)	21 (49%)	
Student	0 (0%)	1 (2%)	
House wife/husband	0 (0%)	1 (2%)	
Other	1 (2%)	3 (7%)	
Geographical area of residence—indices of multiple deprivation (IMD) (grouped):			
Group 1 (most deprived)	22 (51%)	23 (56%)	Fisher's exact *P* = 0.46
Group 2	16 (37%)	18 (42%)	
Group 3	3 (7%)	0	
Group 4 (least deprived)	2 (5%)	2 (5%)	
Type of MS:			
Relapsing remitting	8 (19%)	16 (37%)	Fisher's exact *P* = 0.14
Secondary progressive	19 (44%)	13 (30%)	
Primary progressive	16 (37%)	14 (33%)	
Age at:			
Onset mean yrs, SD	34.3 yrs, 13.9	34.1 yrs, 11.2	*t*-test 0.08, *P* = 0.94
Diagnosis mean yrs, SD	41.4 yrs, 14.3	37.4 yrs, 11.3	*t*-test 1.43, *P* = 0.16
MS duration from diagnosis:			
Mean yrs, SD (range)	16.1 yrs, 11.2 (1.67–50.29)	10.2 yrs, 5.7 (0.89–23.27)	*t*-test 3.01, *P* = 0.003
Median	12.4	9.5	
EDSS score:			
6.0	15 (35%)	10 (23%)	Fisher's exact, *P* = 0.77
6.5	9 (21%)	14 (33%)	
7.0	4 (9%)	6 (14%)	
7.5	3 (7%)	4 (9%)	
8.0	8 (18%)	6 (14%)	
8.5	2 (5%)	1 (2%)	
9.0	2 (5%)	2 (5%)	

**Table 2:** Service use and associated cost (*£*) per 6 months for Black Caribbean and White British groups.

Service	Number of (%) patients using service		Mean (SD) for contacts of the service		Mean (SD) cost ( *£*) for users of specific services only		Mean (SD) cost (*£*) for 43 Black Caribbean and 43 WB patients	
	Black Caribbean	White British	Black Caribbean	White British	Black Caribbean	White British	Black Caribbean	White British
Neurology outpatient^1^	28 (65.1)	29 (67.4)	3 (3.7)	2 (1.1)	461 (616)	231 (187)	289 (535)	145 (186)
Other outpatient use	18 (41.9)	20 (46.5)	3 (2.4)	5 (11.3)	443 (718)	498 (1045)	186 (508)	230 (746)
Nursing/residential home or Hospice	1 (2.3)	1 (2.3)	7 (0)	7 (0)	1059 (0)	1059 (0)	25 (161)	25 (161)
Inpatient services^2^	4 (9.3)	2 (4.7)	14 (9.4)	9 (8.5)	5308 (3529)	3878 (3695)	505 (1843)	180 (1004)
Neurologist	4 (9.3)	0	1.3 (0.5)	0	63 (35)	0	6 (21)	0
General practitioner	31 (72.1)	34 (79.1)	4.2 (6.1)	2.8 (2.4)	157 (211)	101 (115)	113 (192)	85 (111)
Other doctor	2 (4.7)	1 (2.3)	3.5 (3.5)	10	253 (215)	1310	12 (63)	30 (197)
Allied health professionals^3^	30 (70.0)	27 (62.8)	11 (14.0)	16 (10)	352 (472)	471 (746)	264 (435)	296 (630)
District nursing and primary care	31 (72.0)	31 (72.1)	3 (2.5)	9 (22.2)	106 (84.8)	164 (318)	76 (86)	118 (279)
Investigations^4^	26 (60.5)	24 (55.8)	4 (3.9)	29 (1.0)	168 (239)	89 (148)	102 (202)	50 (118)
Formal Home help	18 (41.9)	15 (34.9)	203.7 (207.2)	197.7 (214.4)	7435 (8154)	4957 (4268)	3112 (6378)	1960 (3546)
Informal Care^5^	40 (93.0)	39 (90.7)	36 (59.8)	51 (83.1)	18477 (29231)	24715 (40262)	17188 (28567)	22415 (38979)
Medication	—	—	—	—	—	—	1338 (953)	273 (3060)

^1^Neurology outpatient (neurology outpatient & Day hospital neurology).

^2^Inpatient services (neurology inpatient, intensive care, urology, cardiology, and other inpatient).

^3^Allied health professionals (speech and language therapy, physiotherapy, occupational therapy, social work, and dietician).

^4^Investigations (MRI, CT scans, respiratory function tests, blood tests, and other investigations).

^5^Informal care (personal care support, help with medication, help inside home, help outside home, time on call, and other).

**Table 3:** Bootstrap regression of cost between ethnic groups.

Variable	Reps.	Observed	Bias	Std. err.	[95% conf. interval]		
Ethnicity	1000	−2591.86	−15.02	7484.53	−17279.06	12095.34	(N)
					−17076.30	11754.77	(P)
					−16819.41	12259.80	(BC)

Constant	1000	25777.51	−46.89	5887.20	14224.82	37330.19	(N)
					15310.83	37573.73	(P)
					15486.77	37750.72	(BC)

Note: N = normal.

P = percentile.

BC = bias corrected.

Bootstrap statistics: number of obs. = 86, replications = 1000.

**Table 4:** Bootstrap regression with additional potential predictor variables.

Variable	Reps.	Observed	Bias	Std. err.	(95% conf. interval)		
Time with disease yrs	1000	−542.0193	−7.667818	344.3306	−1217.714	133.675	(N)
					−1236.73	124.6862	(P)
					−1243.697	117.6147	(BC)

EDSS scores	1000	5985.081	51.63416	2951.37	193.4844	11776.68	(N)
					396.7714	11991.88	(P)
					299.2502	11978.44	(BC)

IMD scores	1000	.5103984	.0567446	.8758165	−1.208252	2.229049	(N)
					−1.052415	2.274935	(P)
					−1.136945	2.226263	(BC)

Gender	1000	148.7712	−335.9037	8453.865	−16440.6	16738.14	(N)
					−16866.32	15346.41	(P)
					−16887.99	15305.67	(BC)

Ethnicity	1000	−804.5152	−6.846454	705.3772	−2188.706	579.6757	(N)
					−2183.026	556.8843	(P)
					−2177.213	559.2528	(BC)

Type of MS	1000	1714.387	191.9886	5750.809	−9570.664	12999.44	(N)
					−9150.42	13923.62	(P)
					−9373.646	13810.63	(BC)

Age at interview	1000	−392.8838	−2.470308	369.3031	−1117.583	331.8151	(N)
					−1158.42	331.5103	(P)
					−1165.851	314.4072	(BC)

Constant	1000	20394.31	−227.1215	26323.28	−31260.95	72049.57	(N)
					−31201.81	75481.04	(P)
					−26689.37	78229.74	(BC)

Note: N = normal.

P = percentile.

BC = bias corrected.

Bootstrap statistics: number of obs. = 86, replications = 1000.
